# Increasing Public Health Mosquito Surveillance in Hidalgo County, Texas to Monitor Vector and Arboviral Presence

**DOI:** 10.3390/pathogens10081022

**Published:** 2021-08-13

**Authors:** Clarissa D. Guerrero, Steven Hinojosa, Diana Vanegas, Niko Tapangan, Matthew Guajardo, Sara Alaniz, Narda Cano, Christopher J. Vitek, John Thomas, Valerie Hernandez, Juan Garcia, Bethany G. Bolling, Whitney A. Qualls, Ronald Tyler, Eduardo Olivarez

**Affiliations:** 1 Hidalgo County Health and Human Services, Edinburg, TX 78542, USA; Clarissa.guerrero@hchd.org (C.D.G.); Steven.Hinojosa@hchd.org (S.H.); diana.vanegas@hchd.org (D.V.); NIKO.TAPANGAN@HCHD.ORG (N.T.); matthew.a.guajardo1995@gmail.com (M.G.); Smalaniz49@gmail.com (S.A.); Nzcano@yahoo.com (N.C.); Eduardo.Olivarez@hchd.org (E.O.); 2 Center for Vector-Borne Diseases, University of Texas Rio Grande Valley, Edinburg, TX 78539, USA; john.thomas@utrgv.edu (J.T.); valerie.hernandez01@utrgv.edu (V.H.); juan.garcia02@utrgv.edu (J.G.J.); 3 Texas Department of State Health Services, Austin, TX 78714, USA; Bethany.Bolling@dshs.texas.gov (B.G.B.); wqualls@amcdfl.org (W.A.Q.); Ronald.Tyler@dshs.texas.gov (R.T.)

**Keywords:** epidemiology, Zika virus disease, disease vectors, socioeconomic factors, public health surveillance

## Abstract

From 2016 to 2018, Hidalgo County observed the emergence of Zika virus (ZIKV) infections along with sporadic cases of Dengue virus (DENV) and West Nile virus (WNV). Due to the emergence of ZIKV and the historical presence of other mosquito-borne illnesses, Hidalgo County obtained funding to enhance mosquito surveillance and educate residents on arboviruses and travel risks. During this time period, Hidalgo County mosquito surveillance efforts increased by 1.275%. This increase resulted in >8000 mosquitoes collected, and 28 mosquito species identified. *Aedes aegypti, Ae albopictus* and *Culex quinquefasciatus* made up approximately two-thirds of the mosquitoes collected in 2018 (4122/6171). Spatiotemporal shifts in vector species composition were observed as the collection period progressed. Significantly, temperature variations (*p* < 0.05) accounted for associated variations in vector abundance, whereas all other climate variables were not significant.

## 1. Introduction

*Aedes aegypti* and *Aedes albopictus* are considered the primary vectors for Zika (ZIKV), dengue (DENV) and chikungunya (CHIKV) viruses. Local transmissions of DENV, CHIKV, ZIKV and West Nile virus (WNV) has been documented along the Texas–Mexico border [1,2,3]. These arboviruses are a threat to public health and the economy in areas where these outbreaks occur [4,5]. For example, from 2002–2011, a total of 2274 WNV cases were reported in Texas, with a 6% fatality rate and an estimated economic cost to the state of $112 million [6]. Historically, small outbreaks of DENV have occurred along the Texas–Mexico border and from 2010–2017, 24 locally acquired and 267 travel-associated cases were reported statewide [7]. The risk for DENV transmission is highest in the southernmost Texas counties, with seroprevalence rates for DENV antibodies estimated to be 39% in Brownsville, Texas, based on an epidemiologic survey conducted in 2005 [8]. The prevalence and spread of arboviruses are attributed to an increase in global migration, urbanization, poverty and climate change, factors that specifically impact South Texas [1,5,9,10]. 

From 2012 to 2015, Hidalgo County, Texas, documented locally acquired cases of WNV, DENV and CHIKV, and in 2016, ZIKV infections were identified for the first time [11]. Prior to 2016, Hidalgo County lacked extensive capacity for mosquito surveillance. Sporadic mosquito surveillance efforts were conducted in response to community complaints of high mosquito activity in neighborhoods, which yielded limited data. County surveillance efforts gradually increased as a mitigation strategy to survey the area for mosquitoes; albeit, seasonal and inconsistent. In 2017, Hidalgo County secured supplementary funding to address the ZIKV threat, which generated staff positions and training to support increased mosquito surveillance activities. During the same year, Hidalgo County had the majority (4 of 7) presumed local mosquito-borne cases of Zika virus disease cases within the United States. In 2018, Hidalgo County mosquito surveillance approach strategies were modified to focus on education, data collection and research. Due to the recent ZIKV activity, Hidalgo County increased and expanded mosquito surveillance efforts to protect and inform residents about ZIKV. The primary objectives of this study are to report on mosquito surveillance efforts in the area, characterize local mosquito populations and assess potential associations between climate and mosquito species abundance in Hidalgo County. 

## 2. Results

During the study period, a total of 1207 traps were set, with five total collection methods employed: BG-Sentinel (n = 502), Backpack Aspirator (n = 398), Light (n = 239), Gravid (n = 59) and Fay-Prince (n = 9). A total of 1058 mosquito pools were tested for arboviruses. A total of 8290 female mosquitoes were collected, consisting of 28 different species. Testing was conducted on important vector species by the University of Texas–Rio Grande Valley and the Texas Department of State Health Services. All mosquito pools tested during this time-period were negative for any of the tested viruses (ZIKV, CHIKV, DENV, WNV, St. Louis encephalitis virus and western equine encephalitis virus). 

**Figure 1:**
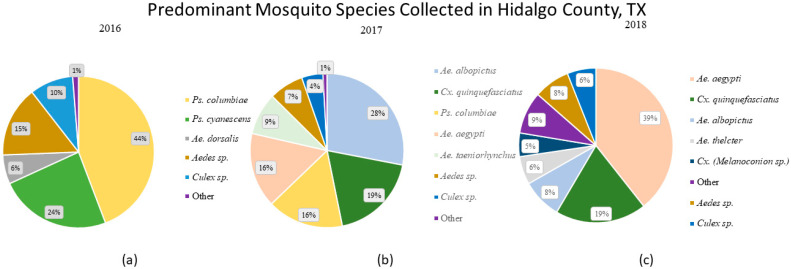
Year 1 (2016), Year 2 (2017) and Year 3 (2018) most common mosquitoes collected are shown, listed in order on each pie chart from highest to lowest abundance. Each color represents a different common species and depicts a shift in relative abundance over time. (**a**) *Ps. columbiae* and *Ae. dorsalis* comprised 68% of total mosquitoes collected (n = 484) for year 1. (**b**) In year 2.79% of the mosquito population was comprised of *Ae. albopictus*, *Ps. columbiae*, *Cx. quinquefasciatus* and *Ae. aegypti*. (**c**) In year 3, two-thirds of the mosquito population consisted of *Ae. aegypti* (n = 2429) and *Cx. Quinquefasciatus* (n = 1475). Figure 1a–c from left to right. Abbreviations: *Aedes* (Ae), *Anopheles* (An), *Culex* (Cx), *Mansonia* (Ma), *Psorophora* (Ps), *Uranotaenia* (Ur).

In 2016, successful collections were conducted from May through to December (Figure 1a). There were 19 species identified, with 68% of mosquitoes consisting of *Psorophora. columbiae* and *Ps. cyanescens*. The remaining 32% of species collected consisted of *Ae*. *dorsalis* (6%), other *Aedes* spp. (15%), other *Culex.* spp. (10%) and other species (1%). In 2017, collections were performedfrom June to December (Figure 1b). A total of 19 species were identified, with the most abundant mosquito species collected being *Ae. albopictus* (28%), *Cx. quinquefasciatus* (19%), *Ps. columbiae* (16%), *Ae. aegypti* (16%), *Ae. taeniorhynchus* (9%), other *Aedes*. spp. (7%), other *Culex.* spp. (4%) and all other species (1%). In 2018, successful trapping collections took place between January and December, with 24 species identified, where 45% of mosquitoes consisted of *Ae. aegypti*, *Cx. quinquefasciatus* (22%), *Ae. albopictus* (10%), *Ae. thelcter* (7%), other species (10%) and *Cx. melanoconion* (6%) (Figure 1c). Collections in 2018, which revealed the highest numbers for *Ae. aegypti* and *Cx. quinquefasciatus* compared to previous years. 

Furthermore, the following species were the most prevalent and consistently present throughout the collection period: *Ae. aegypti*, *Ae. albopictus*, *Ae. thelcter*, *Cx. (Melanoconion)* sp., *Cx. quinquefasciatus*, *Ps. columbiae* and *Ps. cyanscens.* These seven species represented 84% of total mosquitoes collected. As shown in Table 1, the total number of *Ae. aegypti* collected increased (186.8 times) from 13 mosquitoes caught in 2016 to 2429 mosquitoes in 2018 compared to the other common mosquito species over the three-year period. Additionally, in 2018, *Ae. aegypti* was present 11 of 12 months, and *Ae. albopictus* 9 out of 12 months. While we are unable to statistically compare the trapping methods to each other due to varying deployment of traps (both in terms of site and number of times utilized), it is possible to conduct a preliminary comparison of the trapping data. The following trapping methods provided the greatest to least diversity of species caught; CDC light traps (22 species), backpack aspirator (21 species), BG Sentinel 2 traps (19 species), CDC gravid traps (17 species) and Fay-Prince trap (5 species). Through targeted deployment of urban BG sentinel trapping, targeted mosquito surveillance for *Aedes* species mosquitoes resulted in a greater proportional yield of *Ae. aegypti* and *Ae. albopictus*, from less than 1% in year 2016 to 67% in year 2018 (Table 1). 

**Figure 2:**
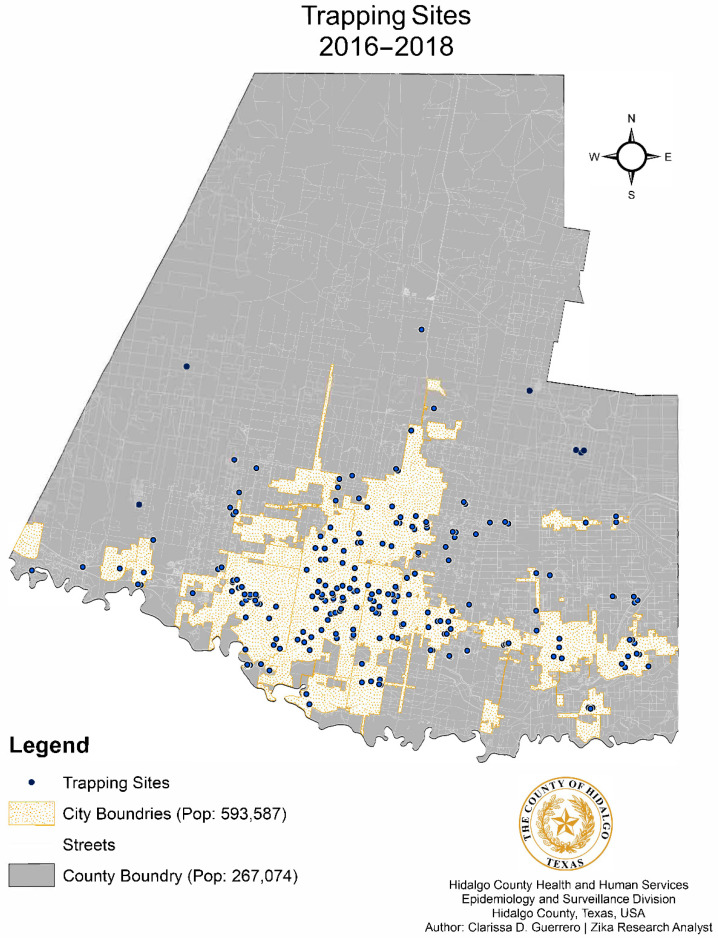
Trap sites are indicated by blue dots on the map. Locations are shown county wide which include city and rural areas. A total of 475 sites were utilized over the collection period.

**Figure 3:**
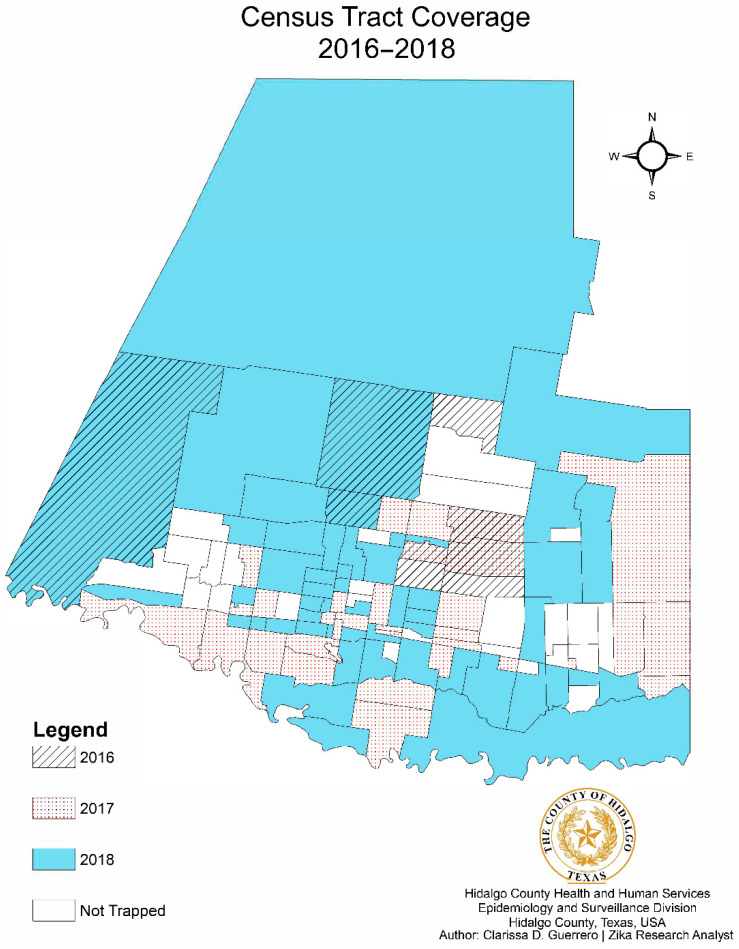
Census tracts where one female mosquito was successfully collected are shown. Tracts with multiple overlays indicate successful trapping over multiple years.

**Figure 4:**
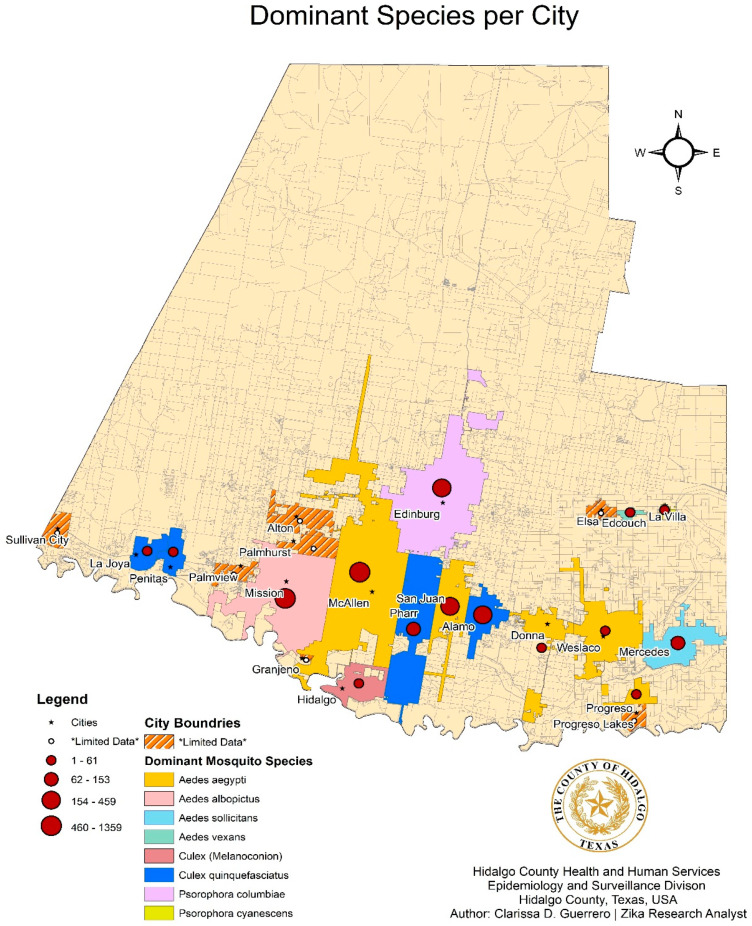
City boundaries are shown on the map and highlighted by the color that represents the dominant species found in that city over the three-year collection period. The size of dot indicates the quantity of mosquito. Cities with striped, orange overlay indicate limited data collected or no dominant species identified due to equal presence of multiple mosquito species.

**Figure 5:**
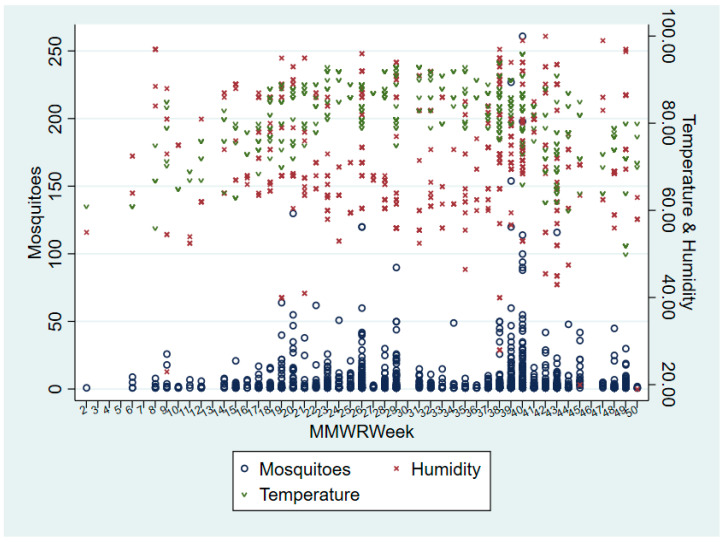
Mosquito counts per MMWR week are shown in relation to temperature °F and humidity levels. Mosquitoes are represented by an open circle, humidity by a red × and temperature by a green v. Scatter plot results depict an increase in mosquitoes as temperature and humidity increased. Results also illustrate mosquito activity at cooler temperatures but not lower than 40 °F.

Trapping sites from 2016 to 2018, and the relation to city limits, are depicted in the form of a dot density map, as shown in Figure 2. The number of census tracts visited increased from 9 tracts in 2016, to 32 in 2017, to 80 in 2018, resulting in an overall 63.4% increased coverage (Figure 3). Census tracts with multiple layers shown indicated successful collections of one or more years denoted. A choropleth map color coded with the dominant mosquito species for each city within the county was also developed with mosquito data (Figure 4). The relative population size of each city is indicated in this map by the size of the circle representing the city. *Aedes aegypti* was identified as the dominant species in 5 of the 22 cities, as illustrated in gold. *Culex. quinquefasciatus* was the dominant species in 4 cities, all illustrated in dark blue. The city of Mission was the only municipality to have *Ae. albopictus* as the dominant species. Baseline trapping locations increased each year from 2016 (n = 1) to 2017 (n = 5) to 2018 (n = 10). A scatter plot was also developed to assess the relationship between mosquitoes and climate variables per Morbidity and Mortality Weekly Report (MMWR) week from 2016–2018 (Figure 5). As shown in Figure 5, elevated temperatures and humidity facilitated the increase of overall mosquito activity. Additionally, it appears that mosquito activity continued to occur during cooler temperatures, however this was not observed below 40 °F.

Linear regression was performed on mosquito counts per trap-night and compared to climate variables, including temperature, humidity, wind and barometric pressure. Results indicated that humidity, wind and barometric pressure did not significantly influence mosquito abundance over the three-year period; however, temperature did significantly influence mosquitoes per trap-night (*p* < 0.01). 

## 3. Discussion

Prior to 2016, cases and outbreaks of Zika were documented in Mexico, Central America and South America [12]. With the risk of vector-borne disease and documented evidence of DENV and ZIKV transmission in Hidalgo County, mosquito surveillance was a vital public health function to monitor disease risk. This study represents a retrospective data analysis of mosquito surveillance activities in Hidalgo County, Texas, 2016–2018. A total of 1207 traps were set, and 8290 female mosquitoes caught. These consisted of 28 different species and resulted in 1058 mosquito pools being tested for arboviruses. All testing results were negative. The surveillance efforts were led by the county health department (Hidalgo County Health and Human Services), so trapping methods and locations were public health-focused and included sentinel sites, case investigation sites and public health intervention sites. Ideally, for a study design, all trapping locations would be the same for the entire collection period, however, due to the county public health response needs, variable trapping methods and locations were employed each year. 

Final surveillance outcomes show an increase of 1.640% for number of traps set in 2018 compared to 2016, resulting in a 1.275% increase in mosquitoes. This increased trapping also allowed mosquito absence data collection, when traps were set, and no mosquitoes were collected. Regarding trap comparison, all collection methods except for Fay-Prince (n = 5) caught a variety of species although, the Fay-Prince trapping only occurred in 2018 with a total of nine traps nights. CDC light trap (n = 22) and backpack aspirator (n = 21) methods resulted in the highest number of species collected, compared to all other collection methods, whereas the BG-Sentinel trap (n = 19) resulted in the largest total number of mosquitoes collected. A species composition shift was observed year to year when examining all species collected. More specifically, *Ae.* species surveillance efforts revealed an 18.684% increase in *Ae. aegypti* and an increase from 0 to 513 *Ae. albopictus* mosquitos caught from 2016 to 2018, with the primary increase in *Ae. aegypti* in 2018 and the primary increase in *Ae. albopictus* occurring in 2017. Although the same number of species were collected in 2016 and 2017, the data from 2016 were dominated by the two *Psorophora* (*Ps.*) sp. which together comprised over two-thirds of the 2016 collections that year. Collection methods and trap locations can be biased for certain mosquito species. The increase in vector mosquitoes during the study period could be attributed to these biases or to other underlying factors, as interpretation of data were based on count per trap, rather than cumulative totals. 

An analysis on climatic factors in relation to general mosquito abundance was performed using linear regression modeling. The low adjusted r squared value (Table 2) may indicate other variables not included in the model that may influence mosquito abundance. Alternatively, there may simply be a high level of variability in mosquito abundance that is only marginally influences by temperature. 

Some limitations encountered throughout the study were limited baseline trapping data, and limited staff and equipment. From a public health approach, it was difficult to establish consistent trapping locations because initial trapping efforts were conducted in response to community complaints. In addition, limited resources (personnel and the traps themselves) did not allow for a full factorial design to compare traps to each other. As a result, trap locations and trapping effort differed between years, making it difficult to compare the spatial and temporal dynamics of mosquito species populations from year to year. In 2016, there were very few locations where trapping took place more than five times. In addition, mosquito trapping only took place primarily from May to October. After limited collections in 2016, trapping increased during 2017 and 2018 when more personnel were hired for mosquito surveillance and Zika response efforts. 

Another complication was collecting weather data for sites. Climate data were primarily recorded at time of collection, however, some data reflected data from historical data sets due to missing data at the time of collection. Furthermore, it was determined that precipitation data collected were not a good variable due to the data collection method, which reflected total rainfall accumulation on the day of collection, resulting in many days with zero rainfall. Therefore, data reflecting one week of prior precipitation, rather than daily rainfall, may provide better analysis and insight. The challenge of collecting weather data also resulted in utilizing historical datasets online. In future surveillance projects, the utilization of stationary weather sensors at each site would address each of these problems and assist in providing more accurate data for climate analysis. 

Vector surveillance programs are critical for informing vector control activities for local public health departments. Integrated vector management (IVM) is the decision-making process for the efficient use of vector control resources to reduce or stop vector-borne pathogen transmission. Screening of mosquito pools for arboviruses is a commonly used practice to gauge the potential threat of mosquito-borne diseases in a community to inform IVM decision processes. With the rising trend in human cases of vector-borne diseases in the United States [13] and recent introductions of newly emerging arboviruses in Texas, maintaining ongoing mosquito surveillance activities is vital. 

Collaborations between multiple municipalities and universities on mosquito surveillance activities provided for creative opportunities to overcome obstacles and expand surveillance efforts during the study period. Involving students from local higher education institutions to assist in county vector surveillance efforts can provide support to health department staff, while providing public health experience to students. By creating these collaborative efforts, the community is served while training future public health frontline workforce. This type of collaborative surveillance is essential in areas such as Hidalgo County, where local Zika outbreaks have occurred. With limited mosquito surveillance funding for local health departments, this strategy can be implemented to continue and enhance surveillance operations.

## 4. Materials and Methods

This mosquito surveillance project took place in Hidalgo County, along the Texas–Mexico border. Hidalgo County is the 7th most populated county in Texas with 860,661 residents and 547.9 persons per square mile [14]. The summer season is hot lasting approximately 4 months, with average temperature highs at 97 °F, whereas winters are typically short and mild with average temperature lows at 52 °F, and average RH of 97% in the summer and 9% in the winter [15].

Vector and climate data were collected from 1 January 2016 to 31 December 2018 with the assistance of Alamo, Hidalgo, La Villa, McAllen, Weslaco, Mission and Pharr municipalities. Mosquito surveillance from 1 January 2016 to 31 May 2017 primarily focused on sporadic trapping based on residential mosquito complaints. Trapping efforts from 1 June 2017 to 31 December 2018 included expanded activities to examine sentinel sites, conduct public health response operations and general surveillance to monitor vector abundance in Hidalgo County. 

Mosquito collections were conducted using BioGents (BG) Sentinel 2 Traps (BIOGENTS^™^, Regensburg, Germany) baited with BG lures, Improved Prokopack Aspirators (John W. Hock Company^©^, Gainesville, FL, USA), CDC Miniature Light Traps (John W. Hock Company^©^), CDC Gravid Traps (John W. Hock Company^©^, Gainesville, FL, USA) and CDC Fay-Prince Traps (John W. Hock Company^©^, Gainesville, FL, USA). Dry ice was used as an attractant for all trap types when available. Supply constraints for dry ice results in the supplier not always having it in stock. Trap type and locations were determined by factors, such as habitat type, accessibility (public/private property), target mosquito populations, detected arboviral disease cases and climate. Traps utilized for collection varied based on personnel available and, traps available at the time of collection.

A total of 475 sites were utilized over the entire trapping period. In most cases, sites were utilized multiple times, but some sites were collected at very infrequently (in some cases, just once). Field locations were a mix of urban and rural sites, most of which were residential. Selection of sites was based on nuisance calls, previous history of mosquito activity and convenience of access. Trapping was also planned for locations where mosquito-borne diseases in humans were detected, but no such locations were identified during this collection period. Traps were left at locations for approximately 24 h before samples were collected. Backpack aspirators were used in early- or mid-morning collections at trap sites to collect samples of resting mosquito populations. Backpack aspirating consisted of a five-to-ten-minute sweeping pattern in grassy or damp areas where trapping occurred. Upon collection, mosquitoes were either sent to the University of Texas Rio Grande Valley (UTRGV) or the Texas Department of State Health Services Arbovirus Laboratory (DSHSAL) for species identification and arbovirus testing. Mosquitoes were identified to species using standard taxonomic keys [16] and sorted by date of collection, trap location, trap type and sex. Some mosquitoes were unable to be identified due to damage during collection. Female vector species were pooled into groups of 1–50 mosquitoes per tube and stored at −80 °C until tested. Both organizations tested blood fed and non-blood fed female mosquitoes for arboviruses and tabulated male counts. Testing at UTRGV included a real-time RT-PCR assay [17] for the detection of ZIKV and DENV (1–4 serotypes) from *Ae. aegypti* and *Ae. albopictus* pools. Testing at the Texas DSHSAL included multiplex real-time RT-PCR assays for the detection of WNV, St. Louis encephalitis virus (SLEV) and Western equine encephalitis virus (WEEV) [18] from *Culex* vector species mosquito pools and ZIKV, CHIKV and DENV (1–4 serotypes) [17] from *Ae. aegypti* and *Ae. albopictus* mosquito pools. All primer and probe sequences are available upon request. 

Weather data were also recorded on the day of trap collection from Weather Underground (www.wunderground.com, 7 February 2020). Less than 1% of the weather data was recorded using a handheld anemometer. Weather data from the nearest larger city was used for smaller cities when data were not available. 

Descriptive statistics were used to evaluate mosquito species composition in Hidalgo County. Due to different reporting methods for male mosquitoes used by UTRGV and the DSHSAL, male species counts were excluded from species composition data analyses. Female mosquitoes were tabulated per species and trap type for each year. Linear regression was conducted to evaluate the relationship between mosquito abundance and climate variables. Additionally, spatial analysis was also conducted on these data using ArcGIS, including a dot density map layered with city and county boundaries to depict trapping locations (Figure 2) and choropleth map to depict census tracts (Figure 3) and dominant mosquito species per city (Figure 4). In addition to ArcGIS, the following software was used for statistical data analysis: StataIC (v15.1, 64-bit, College Station, TX, USA), Epi-Info (v7.2.2.6, Centers for Disease Control and Prevention, CDC, Atlanta, GA, USA) and Microsoft Excel.

## 5. Conclusions

Overall, during the study period, Hidalgo County mosquito surveillance improved with the assistance of funding for program expansion and through multiple collaborations. The community, municipalities and partners benefited from the increased surveillance activities and the collaborations. In addition, an improved understanding of vector species presence and abundance in the county was developed. This project successfully identified predominant mosquito species in the area and examined the potential risk of local disease transmission within vector populations. This information can assist public health response efforts during times of local arbovirus transmission, disease outbreaks and severe weather events. Although there were no virus-positive mosquito pools detected during the study period or during subsequent years (2019–2020), maintaining surveillance in Hidalgo County is critical with continued reports of locally acquired human arbovirus infections. In 2019, two cases of DENV were reported and in 2020, five cases of DENV, one SLEV case and one WNV case (all locally acquired) were reported in Hidalgo County [11]. 

In the future, Hidalgo County plans to establish more baseline sites to continue monitoring mosquito species composition and collect consistent data. Future goals include increasing trapping frequency during off-season months to better depict *Aedes* sp. activity, especially since historical outbreaks of DENV and ZIKV have occurred in the late fall. The need for increased surveillance resources and personnel are essential in Hidalgo County for the continuation of monitoring target vector species. Stable funding for local health departments is critical to ensure that mosquito surveillance activities can continue to monitor disease threats within their respective communities. With health departments playing key roles as frontline responders in public health crises, data collection is key for vector mosquitoes and potential outbreak detection. Continued collaboration between public health, municipalities and higher education institutions can improve the detection, response and intervention of future arboviral risks to the community. 

## Figures and Tables

**Table 1:** Mosquito counts by species and trap type in Hidalgo County. Species totals are shown per year (left) and per collection method (right). A dash indicates no data for that year or species. In total, 28 species were identified over the collection period. Fay-Prince trap data not shown due to limited usage and data collected.

Mosquito Species N = 28	Mosquitoes Caught Per Year				Mosquitoes Caught Per Collection Method			
	2016	2017	2018	Total	BG-Sentinel	Light	Aspirator	Gravid
*Ae. aegypti*	13	285	2426	2724	2015	303	378	28
*Ae. albopictus*	-	421	513	934	623	4	305	2
*Ae. bimaculatus*	-	-	1	1	-	-	-	1
*Ae. dorsalis*	30	-	-	30	-	30	-	-
*Ae. nigromaculis*	-	5	-	5	-	-	5	-
*Ae. sollicitans*	14	63	191	368	20	112	231	5
*Ae. taeniorhynchus*	16	133	127	276	46	7	209	14
*Ae. thelcter*	24	5	361	390	200	144	37	9
*Ae. triseriatus*	1	-	-	1	-	1	-	-
*Ae. vexans*	5	18	138	161	61	54	46	-
*Ae. zooosophus*	-	18	8	26	24	2	-	-
*An. crucians*	2	-	3	5	-	4	1	-
*An. pseudopunctipennis*	2	1	64	676	53	9	5	-
*An. quadrimaculatus*	1	-	5	6	3	2	-	1
*Cx* *. (Melanoconion)*	4	1	304	309	66	207	2	34
*Cx* *. coronator*	20	49	117	186	35	70	44	37
*Cx* *. erraticus*	-	-	58	58	57	-	1	-
*Cx* *. interrogator*	-	2	84	86	26	27	13	20
*Cx* *. nigripalpus*	4	11	97	112	27	53	16	16
*Cx* *. quinquefasciatus*	13	282	1174	1469	797	121	236	315
*Cx* *. restuans*	-	-	9	9	-	-	2	7
*Cx* *. salinarius*	3	3	3	9	4	5	-	-
*Cx* *. tarsalis*	1	1	1	3	-	2	1	-
*Ma. titillans*	2	-	-	5	-	-	-	2
*Ps. ciliata*	-	2	4	6	3	-	2	1
*Ps. columbiae*	212	241	224	677	28	559	75	15
*Ps. cyanscens*	116	10	241	367	32	136	178	21
*Ur. iowii*	-	-	3	3	-	2	1	-
Total mosquitoes caught	583	1551	6156	8290	4120	1854	1788	528
Average count per Collection	-	-	-	-	8.11	7.37	4.49	9.02

**Table 2:** Linear regression for weather variables. Degrees of freedom were 1058 for each test.

Source	Adjusted *r*^2^	*p* Value
Temperature	0.0128	0.0001
Humidity	0.0016	0.1039
Wind speed	0.0066	0.0046
Pressure (HG)	−0.0008	0.7237

## Data Availability

Data supporting reported research results are maintained and available through the Hidalgo County Health and Human Services Department and the University of Texas Rio Grande Valley.
